# Supplementary material for: Are carbon emissions trading and green financial instruments synergistic? -Comprehensive quantitative research based on content analysis
Source: PLoS One. 2024 Mar 7;19(3):e0298601. doi: 10.1371/journal.pone.0298601 (PMC10919601; doi:10.1371/journal.pone.0298601)
Supplement: S1 Data — (ZIP) [file pone.0298601.s001.zip › Data/地级市Z_F工作报告.zip!/╝¬┴╓/╦─╞╜╩╨/└┤╫╘╣1⁄2╓┌║┼í╛┬φ┐╦╩2╛▌═°í┐_2005.docx]

各位代表：

　　现在，我代表四平市人民政府，向大会作政府工作报告，请予审议。

　　一、2004年工作回顾

　　2004年，新一届政府在市委的正确领导下，团结和带领全市人民，紧紧抓住振兴东北老工业基地和扶持粮食主产区的战略机遇，以经济建设为中心，全面实施项目带动战略，齐心协力，埋头苦干，各项工作全面推进，实现了良好开局。全市生产总值276.3亿元，同比增长15.1%；一般预算全口径财政收入16.55亿元，按可比口径计算增长12.4%；全社会固定资产投资完成55亿元，同比增长17%；社会消费品零售总额实现86.9亿元，同比增长11.6%。

　　项目建设成效显著。全市共开发建设项目604个，其中投资亿元以上项目30个，投资1000万元以上在建项目249个。争取前两批老工业基地调整改造项目15个，基础设施等国债项目30项，共争取国家无偿补助资金5.72亿元。实施较大技改项目75项，完成投资19.4亿元，同比增长31%。12户制药企业通过国家GMP认证。

　　国企改革积极推进。全市应深化改制的国有企业309户，已经完成和正在实施的171户，占55%；其中市直工业已完成69%的改制任务。完善城镇社会保障体系试点稳步推进。国内知名大企业集团中国昊华、江苏雨润、广东立白、南京大吉分别与四平联合化、市肉联厂、四平汉高和市钢丝绳厂进行资产重组。红嘴集团兼并了市啤酒厂、梨树天龙酿酒厂和淀粉厂，新上了100万吨钢和100万吨水泥项目，实现新的跨越式发展。企业上市工作积极推进，吉农高科公司被省证监局确定为我省首批14户通过上市辅导的公司之一。

　　工业经济增长势头良好。全市规模以上工业企业完成总产值155.9亿元，同比增长24.3%，比全省平均水平高1.9个百分点；实现工业增加值53.5亿元，同比增长19.8%，比全省平均水平高1.2个百分点；实现利润4亿元，同比增长4.5倍，全省增幅第一；工业经济效益综合指数达到128%，比上年提高21个百分点。工业经济结构不断优化，机械制造和农副产品深加工两大主导产业产值已占整个工业产值的51.6%。70户年产值5000万元以上工业企业实现利润占全市工业利润总额的90%，成为左右我市工业经济形势的主导力量。

　　农村经济呈现喜人局面。全面落实中央1号文件和“一免三补”政策，充分调动农民生产积极性，粮食总产量达到115亿斤，创历史最高水平。畜牧业继续保持强劲发展势头，猪牛羊禽饲养量分别达到707万头、232万头、172万只和1.38亿只。防控高致病性禽流感取得全面胜利，实现了“四无”目标。大力发展农产品加工业，黄龙、华正、天龙酿酒、红嘴油脂等一批龙头企业不断发展壮大。农业标准化生产取得明显进展，落实农业标准化小区15个，有18个产品获国家绿色食品认证，85个产品获国家无公害食品认证。农村新型合作经济组织不断涌现，总数达到972个。劳务输出力度不断加大，达36万人（次）。

　　民营经济不断发展壮大。实施政府推动、政策牵动、能人带动、宣传发动，增强了广大干部群众的市场意识、创业意识和致富意识。有近700名机关干部带头离岗创业，广大群众自主创业，有力推动了民营经济的快速发展。全市个体工商业户发展到89974户，同比增长5%；民营企业达到2664户，同比增长6%；从业人员达到33.6万人，同比增长13.9%。营业收入完成255.6亿元，同比增长35.5%。民营经济增加值和上缴税金分别达到71.5亿元和5.4亿元，同比增长45.8%和43.9%。

　　招商引资成效明显。参加和举办了北京科博会、渝洽会、四平?杭州项目推介会和广东?吉林经贸洽谈会等大型招商活动，广交朋友，扩大了四平知名度。全市共引进项目565个，实际到位资金52.1亿元。与国家开发银行建立了金融合作框架，到位基础设施贷款5000万元。新办外商投资企业16户，实际到位资金1108万美元。出口总额2908万美元，同比增长30.8%，创历史最好水平。开发区载体和对外开放作用明显增强。

　　城市建设取得新进展。组织建设一批重点基础设施项目。四平火车站站舍改造工程部分交付使用，站前广场和人防工程投入使用，整个站前区域改造接近尾声。北河护堤工程完成90%，污水截流干管工程完成5.88公里，四平战役纪念馆主体工程完工。新建、翻建英雄大街东段、公园北街等15条街路，维修中央东路、九马路等27条主次街路。南环线建成通车，北环线全面开工。建设公路项目147项，竣工公路里程1135.4公里，村村通油路率达到51.8%。旧城改造保持适度规模，小区建设水平有新的提高。开展“城区环境综合整治百日会战”和6个专项整治战役，环境污染得到有效控制，净化、绿化、亮化、美化水平有所提高，巩固了国家卫生城市成果。

　　各项社会事业协调发展。重视科技成果的转化和应用，申请国内专利82项。完善基础教育，加强农村教育和未成年人思想道德建设，教育教学改革取得明显成效。市县两级疾病预防控制中心全部建成投入使用，公主岭新型农村合作医疗试点工作初见成效。国家农村部分计划生育家庭奖励扶助制度试点市工作得到全面落实。加强文化市场监管，文化艺术活动成果丰硕，四平艺术团赴日本别府市演出取得圆满成功。广播电视事业取得新发展，设备水平和播出质量明显提高。竞技体育成绩显著，在第28届雅典奥运会上我市运动员获得银牌一枚、2人取得第四名；群众性体育活动广泛开展，成功举办了第三届社区体育大赛。国土资源对经济社会发展的作用明显增强。社区建设不断发展。双拥模范城成果进一步巩固。民族宗教、档案、外事、侨务、旅游、地震、气象、地方志和残疾人救助等各项工作也取得了新成绩。群众生活质量和水平不断提高。全市城镇居民人均可支配收入7778元，同比增长15.9%。农民人均纯收入3063元，同比增长20%。新开发就业岗位6.2万个，城镇新就业5.1万人。全市共拨付养老金、失业金5.5亿元，连续三年实现“两个确保”。城镇低保工作扎实开展，低保人数达到10.2万人，发放低保资金8200余万元。对农村特困群众实施了最低生活保障。认真解决拖欠农民工工资问题。加强食品、药品、农资等市场监管。二龙山水库污染综合治理取得初步成果，点源和面源污染得到有效控制，水质已经明显好转。市区有64542户居民享受到了全天供水服务，覆盖面达到80%以上。

　　民主法制和政府自身建设进一步加强。自觉接受人大的法律监督、工作监督和政协的民主监督。全年办理人大代表建议91件，政协委员提案171件，办复率达100%。切实加强安全生产整治和监督力度，全年没有发生重特大安全生产事故。集中处理信访突出问题及群体性事件，解决了一批信访积案，积案息访率达到42%。深入开展严打整治斗争，社会治安形势比较稳定。认真贯彻落实《行政许可法》，取消行政许可项目553项，占原有行政许可项目的68.5%。深入开展政务公开、效能监察和审计监督，坚决纠正部门和行业不正之风，集中解决损害群众切身利益的突出问题，查办了一批违法违纪案件。

　　各位代表，过去的一年，人民群众得到更多的实惠，全面建设小康社会迈出坚实的一步，实现了振兴四平老工业基地的良好开局。在此，我代表市人民政府，向全市广大工人、农民、知识分子和各级干部，向中省直各单位，向驻平部队、武警官兵，向关心和支持四平市建设的港澳同胞、台湾同胞、广大侨胞及各界朋友，表示衷心的感谢！对人大、政协、各民主党派、人民团体、各界人士给予政府工作的有效监督、大力支持和热情帮助，表示衷心的感谢！

　　回顾一年来的工作，我们也清醒地看到，我市经济工作和各项事业中还存在一些矛盾和问题。主要是发展思路不宽，速度不快；经济总量不大，投入相对不足，融资比较困难；市场化程度较低，经济发展的活力和竞争力不强；传统产业比重比较大，技术装备相对落后，各类人才缺乏；全民创业的活力有待进一步激发，民营经济总体上规模小、科技含量低、管理粗放；社会支撑能力不强，财政增长水平不适应经济社会的发展需要，结构性失业压力较大，困难群众和弱势群体生产生活问题比较突出，还潜在着一些不稳定因素；经济发展环境、政府机关的工作作风和办事效率还有待进一步改善。对这些问题，我们一定高度重视，认真加以解决。

　　二、2005年主要任务

　　2005年是我市改革发展进程中十分重要的一年。我们既要全面完成“十五”计划，实现振兴老工业基地的第一步目标，又要谋划制订好“十一五”规划，聚精会神抓经济，千方百计快发展。同时也要看到，外部环境有许多不确定因素，能源、交通运输、资金等仍然紧张，转变经济增长方式的要求更加迫切。特别是在全省各地竞相发展的形势下，进一步加快四平市发展显得尤为紧迫。我们必须紧紧抓住振兴老工业基地这个战略机遇，进一步增强发展意识、竞争意识和危机意识，坚持以经济建设为中心不动摇，坚持走新型工业化道路不动摇，坚持全面实施项目带动战略不动摇，鼓足发展劲头，强化发展措施，使四平经济尽快步入快速健康的发展轨道。今年全市工作的总体思路是：以邓小平理论和“三个代表”重要思想为指导，全面贯彻党的十六届四中全会、省委八届六次全会和市委四届四次全会精神，充分利用好国家振兴东北老工业基地和扶持粮食主产区的各项政策，牢固树立和认真落实科学发展观，以发展为主题，全面实施项目带动战略，着力推进国企改革和对外开放，加快调整经济结构，转变经济增长方式，走新型工业化道路，加大固定资产投入，做大做强主导产业和优势产业，关心群众生活，维护社会稳定，提高工作效率，加快推进四平多产业综合性新型工业城市建设进程。

　　主要预期目标是：全市生产总值增长15%以上，突破300亿元大关；一般预算全口径财政收入增长12%以上；全社会固定资产投资增长50%以上；城镇居民人均可支配收入增长10%以上，农民人均纯收入增长8%以上；城镇登记失业率控制在4%以内；居民消费价格指数为104%；人口自然增长率控制在6‰以内。

　　实现上述目标，要重点抓好以下工作：

　　（一）突出抓好项目建设，加大固定资产投资力度。

　　全力开展项目落实年工作，积极抓好工业项目、国家优质粮食工程项目和能源、交通、水利等基础设施项目以及招商引资签约项目的落实，提高项目的竣工率和达产率。前两批15个老工业基地项目年内全部开工建设。重点抓好今年新确定的50个重点项目建设，加快实施华润黄龙玉米深加工、中国昊华年产5万吨离子膜烧碱、雨润集团年屠宰100万头猪生产线、红嘴集团10万吨变性淀粉、伊通200万吨水泥、双辽10万千瓦风力发电等项目建设；积极推进通钢20万吨冷轧带钢、伊通150万平方米化成箔、万?乳制品加工等续建项目的建设进程；着力做好四平热电厂二期工程、双辽发电厂二期工程和引松入平等基础设施建设项目的前期工作。积极做好第三批30个老工业基地项目的申报工作。

　　（二）全力打好国企改革攻坚战，增强经济发展活力。

　　今年是我市国企改革的关键一年，要集中力量，克难攻坚，全面完成国有企业的改革任务。按照有利于企业发展、有利于维护职工利益、有利于调动改革者积极性的原则，坚持做到整体改制到位、债权债务处理到位、职工劳动关系转换到位、国有资本退出到位，改制后基本建立现代企业制度，为振兴老工业基地再造体制和机制活力。建立和完善组织领导体系和工作推进体系，落实责任和措施，对改制企业进行分类排队，一企一策，逐户制定改制方案，进行审计评估，组织统一挂牌出售和产权招商等，使企业改制工作积极推进、稳步实施、有序进行，全面实施攻坚。加快并轨试点步伐，通过国有股权转让、资产变现、土地置换、引进域外资本、减免有关税费等，千方百计筹足改革成本，保证企业与职工顺利解除劳动关系或终止劳动合同，促进和保障改制工作顺利进行。

　　积极推进集体企业改革。加快建立和完善国有资产监管体系。组织好市县两级政府机构改革和事业单位人事制度改革。

　　（三）坚持走新型工业化道路，努力提高经济运行质量。

　　以提高增长质量和效益为中心，加大调度、监测、协调和服务力度，突出解决好煤、电、油、运、气等紧张问题，支持年产值5000万元以上重点企业增加生产、提高效益。确保全市规模以上工业增加值增长18%以上。大力推进技术进步，加快结构调整，注重节约资源，发展循环经济。在做大做强主导产业和优势产业的基础上，大力发展能源、冶金、建材、纺织、医药和旅游等特色产业，进一步拓宽发展领域。突出大企业的带动作用，形成一批优势产业和中小企业群，提升核心竞争力。深入实施质量兴市活动，不断增强地产品的品牌知名度和市场竞争力。增强金融风险防范意识，提高金融支持经济发展的能力与水平。大力发展市场中介、连锁经营、物流配送、绿色餐饮等服务业，加强市场开拓，扩大消费需求。进一步整顿规范市场经济秩序，开展知识产权、食品药品安全、土地及矿业权市场等专项整治。坚决打击假冒伪劣和经营欺诈行为，加强市场监管，维护企业和消费者权益。加强企业管理，抓好扭亏增盈，提高企业经济效益。大力增收节支，依法加强税收征管，理顺税费收缴秩序，确保财政收入稳步增长。

　　（四）高度重视“三农”问题，推进农村的工业化、城镇化和现代化。

　　实施工业拉动、项目带动、改革推动、城乡互动，不断提升县域经济整体水平。抓好优质粮食产业工程建设，加快已列入国家第一批计划的公主岭、梨树优质专用粮食产业基地建设的步伐，切实做好双辽、伊通争取列入第二批计划的工作，扩大专用玉米、优质水稻、高油大豆种植面积，尽快形成专用粮食优势产业带，提高粮食产业竞争力。积极推进畜牧大市向畜牧强市转变，继续狠抓牧业小区和规模养殖场、养殖户，突出抓好动物疫病防控，把畜牧业发展成农民增收的大产业。大力发展绿色有机农业，推行标准化生产，打造品牌，争创名牌，扩大四平农产品知名度。抓住中央进一步加大农业投入的机遇，积极争取扶持产业化龙头企业、高标准农田建设、水利设施、生态林业、农业机械化、农业标准化、农技推广、农民培训、农业信息网络等方面的项目，为提高农业综合生产能力打下基础。加快推进农业产业化经营，重点抓好立市、立县的农产品加工项目。积极培育农村各类合作经济组织，提高农民市场化组织程度，培育农民创业主体。加大劳务输出力度，加强组织、搞好培训、完善服务，引导农民走出土地、走出农村、走出四平，进一步提高非农收入水平。

　　全面落实好党在农村的各项政策。进一步深化农村税费改革，积极推进乡镇机构改革试点和农垦企业税费改革试点，加快县乡财政管理体制和粮食流通体制改革，建立精干高效的基层行政管理体制。

　　（五）实行鼓励、支持和引导的政策措施，大力促进民营经济发展。

　　树立亲商、富商、安商、扶商观念，营造尊重财富、崇尚创业的致富氛围，通过放宽市场准入条件、简化和规范审批登记手续、实行税收优惠政策、加大财政支持力度、拓宽融资渠道、建立健全服务体系、发展行业协会、鼓励全民创业等，推动民营经济实现30%以上的增长速度。充分发挥我市建材、农产品等方面优势，兴建和培育特色市场，为发展民营经济搭建载体、提供条件。发展壮大重点企业，扶持培育个体工商户和中小民营企业群体。针对沿海发达地区资本流动的新情况，用优惠政策、优良环境、优质服务，吸引大批沿海发达地区企业来四平投资兴业，促进我市经济的快速发展。

　　（六）加快对外开放步伐，加大招商引资力度。

　　全方位、宽领域、深层次扩大对内对外开放，争取完成招商引资80亿元，增长50%。坚持“走出去”和“引进来”相结合，围绕产业基地建设、基础设施、国企改革和发展高新技术产业大力开展招商引资。将台湾、日本、韩国、“长三角”、“珠三角”和环渤海地区等作为招商重点，参加和举办招商活动，力争有新的突破。围绕全省打造“吉林?长春?四平”工业经济带的思路，各县（市）、区要因地制宜地培育和建设1?2个具有地方特色的工业园区。充分发挥开发区的载体功能和集聚效应，加强开发区规划建设，搞好结构调整和优化升级，使开发区成为经济新的增长点。积极实施以质取胜和市场多元化战略，转变出口增长方式，外贸出口增长38%，实际利用外资增长40%。

　　（七）加大城市建设和管理力度，巩固和发展国家卫生城市成果。

　　统筹城市规划、建设和管理，加快中心城市建设步伐，增强城市对经济的带动能力。调整城市空间布局，确定城市发展定位，搞好城市总体规划修编。合理安排房地产开发规模和布局。全面加强基础设施建设。完成北河护堤、迎宾街公铁立交桥、战役纪念馆等续建工程，开工建设北河滨河公园、铁东文化公园、宏泰公园及传染病院迁址工程。大力推进市政道路建设，完善城市交通网络，拓展城市规模。新建平东大路、平东东路、英雄大街西段、六孔桥路，维护维修主次干道20条。继续加大交通设施投入力度，年内完成投资17.1亿元。北环路建成通车，开工建设102线、集锡线、四叶线一级路改建等一批重点工程，建设公路里程1274公里，当年竣工1160公里，村村通油路率达到65%。全面加强城市管理。深入贯彻实施《四平市加强国家卫生城市管理实施意见》，明确划分各部门管理职能，突出两区的主导作用，把各项基础工作落到实处。大力推进绿化进程，继续抓好小区、广场、街路、校园、庭院绿化，建设环城隔离林带。继续推进亮化工程建设，完成“两河”、“四场”、“八街”和30个亮化示范点建设。加大净化、绿化、亮化、美化力度，巩固和扩大国家卫生城市成果，营造清新、优美、舒适的人居环境。

　　（八）统筹兼顾，推进各项社会事业协调发展。

　　全面实施科教兴市战略，促进科技、教育与经济建设紧密结合。加快关键技术和高新技术的开发应用步伐，提高科技、知识产权对经济的贡献率。继续深化教育教学改革，巩固“两基”成果，完善农村教育，积极发展职业教育和民办教育。完成中小学教师专业技术职务“评聘合一”改革试点工作。认真抓好未成年人思想道德建设。大力开发人才资源，积极引进人才、培养人才、使用人才、吸引人才。加大文化市场的监督管理力度，大力发展文化产业。加强公共卫生体系建设，抓好卫生执法监督、疾病预防控制、城镇医疗体制改革和农村卫生工作，进一步完善新型农村合作医疗试点。积极发展广播电视事业，开工建设广电大厦。广泛开展全民健身活动，增强竞技体育综合实力。深化人口和计划生育综合改革，广泛开展计划生育“三结合、五关爱”活动。继续加大二龙山水库污染综合治理力度，提高水质标准，改善生态环境，满足人民群众生产生活用水需要。大力推进社会主义精神文明建设。深入开展“双拥”活动，巩固双拥模范城成果。

　　(九)关心群众生活，营造稳定和谐的社会环境。

　　今年全市确定创造城镇就业岗位5.3万个，城镇新增就业4.2万人。深入开展“创业促就业”活动，增加再就业资金投入，落实好小额担保贷款、主辅分离、安置富余人员等各项扶持政策，实行失业保险、城市居民最低生活保障和再就业联动机制，强化促进就业的功能。加强社会保障体系建设。继续加大社会保险扩面、续保、征缴和清欠的工作力度，做到应保尽保，应收尽收，确保各项社会保险金及时足额发放。不断完善城市低保制度，逐步提高低保补差标准。基本解决拖欠农民工工资问题。全面推进社会救助体系建设，切实解决好困难群众的生产生活问题。

　　努力构建和谐稳定社会，创建“平安四平”。高度重视信访工作，严格落实信访工作责任制和责任追究制，突出解决企业改制、回迁安置、征地补偿、涉法涉诉等涉及群众切身利益的各种突出问题。办好市长公开电话。切实抓好安全生产，加大依法监管和专项整治力度，促进安全生产状况进一步好转。加强社区建设。搞好政务公开、厂务公开、村务公开。始终保持“严打”态势，深入推进社会治安防控体系建设，维护全市政治安定和社会稳定。

　　(十)提高效率,服务经济,建设廉洁高效政府。

　　进一步改进政风，提高政府行政能力。完成今年的各项工作任务，必须切实提高加快发展、协调发展、稳定发展的能力。把握发展全局，进行科学民主决策。既要处理好当前的突出问题和矛盾，又要注意解决好长期性、战略性问题。全面实现“十五”计划，高标准完成“十一五”规划编制工作，谋划好今后发展。倡导开短会、讲短话、发短文，加快工作节奏，提高工作效率，集中精力抓大事、抓工作、抓落实。坚持重实干、讲实话、办实事、求实效，坚决反对形式主义。要把保持共产党员先进性教育活动落实和体现到服务型政府建设上，走出机关，服务基层，解决实际问题，推动和促进发展。

　　进一步转变政府职能，为加快发展创造良好环境。认真贯彻国务院《全面推进依法行政实施纲要》，坚持依法行政、依法决策、依法办事。自觉接受人大、政协和广大群众的监督，认真办理人大代表议案、建议和政协委员提案。支持工会、共青团和妇联等群众组织开展工作。加强对行政执法行为的监督和规范。全面实行“阳光政务”，突出解决集中审批、联合办理、规范服务和信息公开、加强监督等问题。实施行政审批时限制和责任追究制，减少审批环节，简化审批程序，缩短审批时间，提高审批效率。对影响审批时限的，坚决追究当事人和有关领导的责任。进一步落实政府新闻发布和重大决策、重要事项的公开咨询、公开听证及公示等制度，增强政府工作的透明度，提高政府公信力，构筑诚信社会，打造“信用四平”。

　　进一步加强廉政建设，深入开展反腐败斗争。建立健全教育、制度、监督并重的惩治和预防腐败体系。支持监察、审计部门依法独立履行监督职责，严肃查处各种违法违纪案件。认真开展专项治理，坚决纠正部门和行业不正之风。努力建设一支高素质的公务员队伍。

　　各位代表，面对新的形势和任务，我们充满信心，同时也深感责任重大。让我们在市委的正确领导下，带领全市各族人民，解放思想，求真务实，埋头苦干，为早日实现四平老工业基地的振兴而努力奋斗！
